# Global Burden of Alzheimer's Disease and Dementia: Trends in Mortality, Risk Factors and Incidence Across Sustainable Development Index Regions from 1990 to 2021

**DOI:** 10.1002/alz70860_103531

**Published:** 2025-12-23

**Authors:** Muhammad Safiullah, Muhammad Imaz Bhatti, Muhammad Atif Bashir, Muhammad Ali, Anurag Jha, Izza Fatima, Muhammad Nabeel Saddique, Hammad Javaid

**Affiliations:** ^1^ King Edward Medical University, Lahore, Punjab, Pakistan; ^2^ Rawalpindi Medical University, Rawalpindi, Punjab, Pakistan; ^3^ Nibras Research Academy, Lahore, Punjab, Pakistan; ^4^ King Edward Medical Univseristy, Lahore, Punjab, Pakistan

## Abstract

**Background:**

Alzheimer's disease and dementia are major global health concerns, with growing prevalence and significant socio‐economic impact. This study analyzes the global burden of Alzheimer's disease and dementia from 1990 to 2021, focusing on trends in mortality, incidence, and disability‐adjusted life years (DALYs). Using data from the Global Burden of Disease (GBD) study, we assess regional variations across Sustainable Development Index (SDI) regions and identify key risk factors, including elevated fasting plasma glucose and body mass index (BMI).

**Method:**

We used the Global Burden of Diseases Study 2021 to estimate incidence, mortality rate, and disability‐adjusted life years (DALYs) for ADOD in Pakistan. Estimated annual percentage changes (APCs) and average annual percentage changes (AAPCs) were used to quantify the temporal trends from 1990 to 2021.

**Result:**

Globally, the age‐standardized death rate (ASDR) for Alzheimer's disease and other dementias remained constant from 1990 to 2021 (AAPC: 0.004%; 95% CI: ‐0.0072 to 0.0178), with an initial APC increase of 0.09% (1990–1996), a decrease of ‐0.07% (1996–2011), and a rise of 0.07% (2011–2021). Similarly, the age‐standardized DALY rate fluctuated, with an APC of 0.12% (1990–1996), a decline of ‐0.08% (1996–2012), and an increase of 0.11% (2012–2021). The age‐standardized incidence rate (ASIR) also exhibited fluctuations, with a marked rise from 2019 to 2021 (APC: 0.94%). Overall, death and DALY rates are rising globally, with high‐SDI countries experiencing the highest burden, driven primarily by elevated fasting plasma glucose and BMI. While low‐SDI countries show lower rates, the trend remains upward. Across all metrics, females consistently exhibited higher rates than males.

**Conclusion:**

The global burden of Alzheimer's disease and other dementias is steadily rising, with high‐SDI countries bearing the greatest burden due to modifiable risk factors like fasting plasma glucose and BMI. Despite lower rates in low‐SDI countries, the upward trend is evident worldwide, with females consistently showing higher rates across all metrics. These findings underscore the urgent need for targeted prevention strategies and interventions to address this growing public health challenge.

